# Supplementary material for: The aryl hydrocarbon receptor and FOS mediate cytotoxicity induced by Acinetobacter baumannii
Source: Nat Commun. 2024 Sep 11;15:7939. doi: 10.1038/s41467-024-52118-7 (PMC11390868; doi:10.1038/s41467-024-52118-7)
Supplement: Supplementary file 1 — Supplementary Information [file 41467_2024_52118_MOESM1_ESM.pdf]

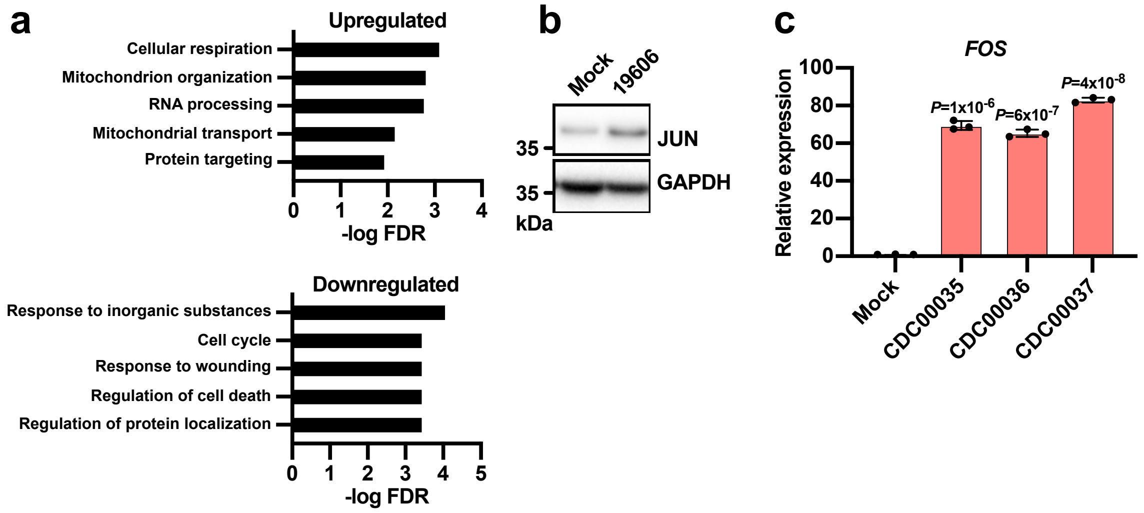

Supplementary Figure 1. Analysis of the host responses towards *A. baumannii* infection, related to Figure 1.

(a) Gene Ontology (GO) analysis of the significantly differentially regulated proteins presented in Figure 1B.

(b) Western blot analysis of A549 cells infected with wild-type ATCC 19606 *A. baumannii* (3 hpi). The experiments were repeated three times with similar results obtained.

(c) Quantification of *FOS* mRNA levels by qRT-PCR in A549 cells infected with *A. baumannii* (3 hpi). n = 3 independent experiments.

Data are presented as mean values +/- SD, P-values by unpaired two-tailed t-test.

Source data are provided in the Source Data file.

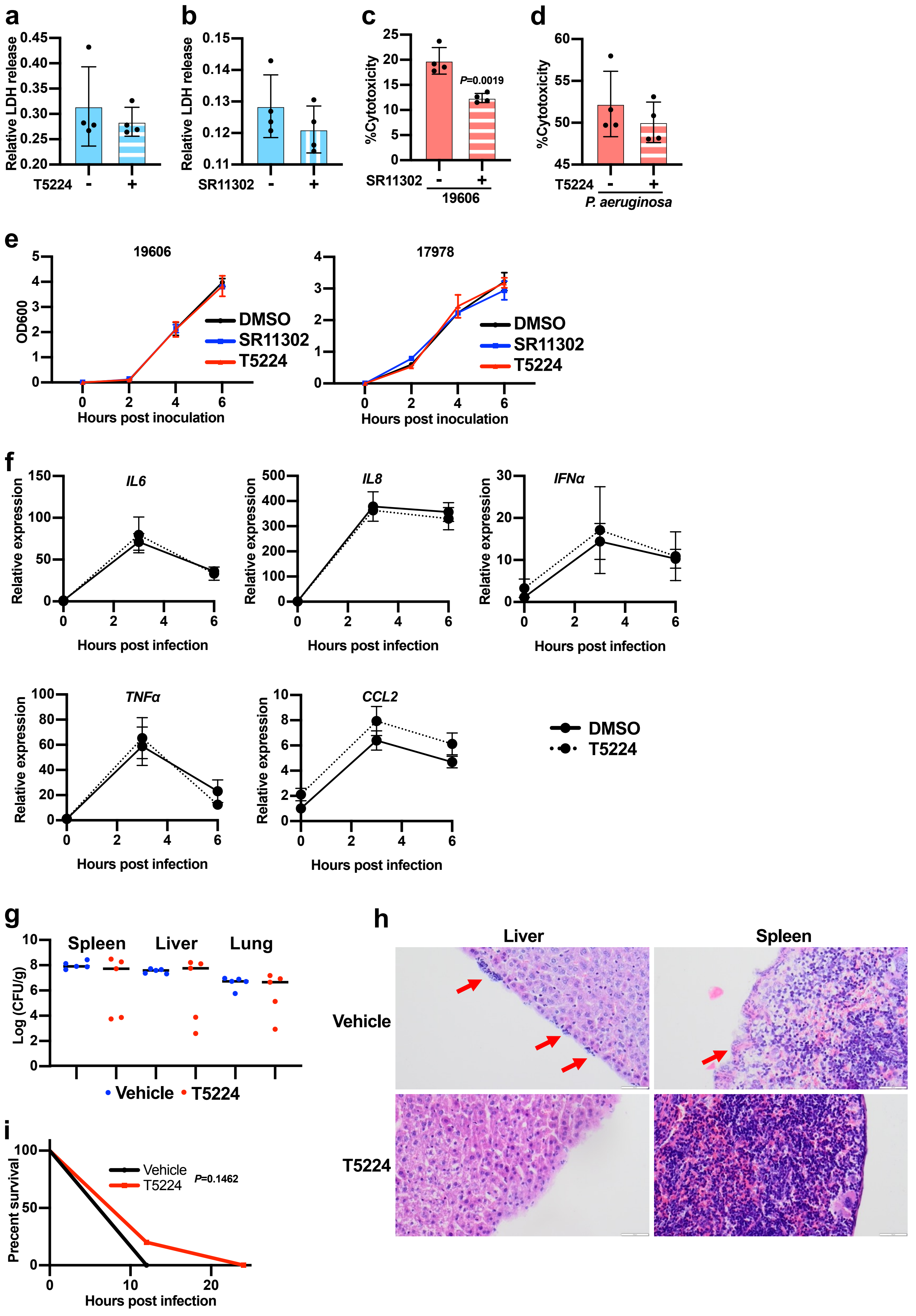

a
Relative LDH release
T5224 - +
b
Relative LDH release
SR11302 - +
c
%Cytotoxicity
P=0.0019
SR11302 - +
19606
d
%Cytotoxicity
T5224 - +
P. aeruginosa
e
19606
OD600
DMSO
SR11302
T5224
Hours post inoculation
17978
DMSO
SR11302
T5224
Hours post inoculation
f
IL6
Relative expression
Hours post infection
IL8
Relative expression
Hours post infection
IFNα
Relative expression
Hours post infection
TNFα
Relative expression
Hours post infection
CCL2
Relative expression
Hours post infection
DMSO
T5224
g
Spleen
Liver
Lung
Log (CFU/g)
Vehicle
T5224
h
Liver
Spleen
Vehicle
T5224
i
Precent survival
Vehicle
T5224
P=0.1462
Hours post infection

Supplementary Figure 2. FOS inhibitors work specifically to reduce cytotoxicity of *A. baumannii* infections, related to Figure 2.

(a,b) LDH release assays of uninfected A549 cells treated with T5224 (100 μM for 24 h) or SR11302 (10 μM for 24 h).

(c,d) LDH release assay of A549 cells infected with *A. baumannii* or *P. aeruginosa* and treated with SR11302 (10 μM for 24 h) or T5224 (100 μM for 24 h) respectively.

(e) *A. baumannii* strains 19606 ($0.5x10^9$) and 17978 ($2.5x10^9$) were inoculated into 3mL of LB media, which were supplemented with T5224 (100 μM), SR11302 (10 μM) or DMSO (solvent control). Optical density (OD600) of the cultures was measured at the indicated time points.

(f) Quantification of *IL6*, *IL8*, *IFNα*, *TNFα* and *CCL2* gene expression by qRT-PCR in A549 cells infected with *A. baumannii* (3 and 6 hpi) and also treated with DMSO (solvent control) or T5224 (100 μM). n = 3 independent experiments.

(g) Bacterial burden of different organs from mice infected with *A. baumannii* (8 hpi) following the protocol shown in Figure 2G. n=5 animals.

(h) Histological analysis of the livers and spleens from the mouse infection model described in Figure 2G. The slides were stained with hematoxylin and eosin (H&E) for visualization. Red arrows indicate aggregates of fibrin and neutrophiles resembling an acute peritonitis.

(i) Survival curve of mice infected with *A. baumannii* clinical isolate FDA-CDC AR-BANK#0280 with or without T5224 treatment. n=10 animals. P-value was calculated using Gehan-Breslow-Wilcoxon test.

(a-f) Data are presented as mean values +/- SD.

(a-d) Cells were seeded in 4 different wells per group, Treatment and measurement were performed independently for each well. Experiments were repeated independently three times and similar results were obtained.

Source data are provided in the Source Data file.

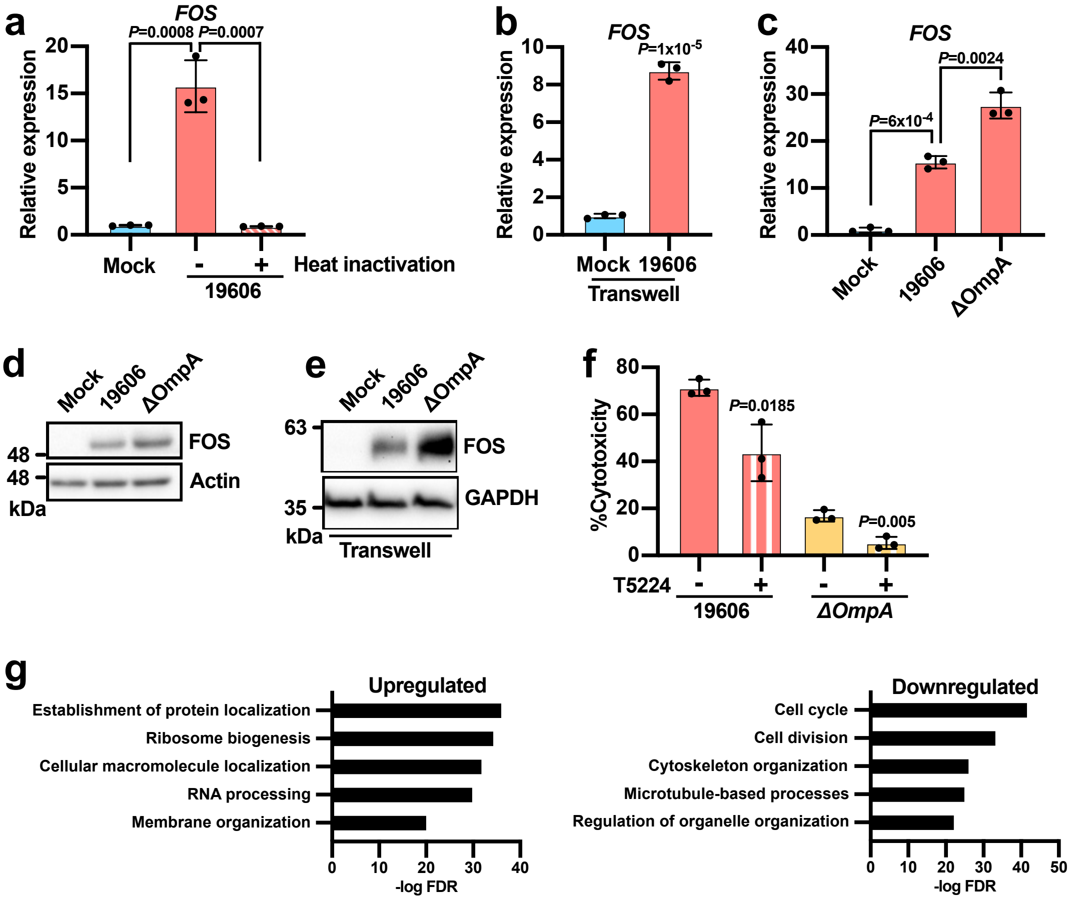

Supplementary Figure 3. Analysis of the host responses towards OMVs, related to Figure 3.

(a-c) Quantification of *FOS* transcript levels by qRT-PCR in A549 cells infected with wild-type *A. baumannii* ATCC 19606 or the *ΔompA* mutant (3 hpi). In panel B, the bacterial cells were physically separated from the A549 cells using a transwell system. n = 3 independent experiments.

(d) Western blot analysis of A549 cells infected with wild-type ATCC 19606 or the *ΔompA* mutant of *A. baumannii* (3 hpi).

(e) Western blot analysis of A549 cells infected with wild-type ATCC 19606 or the *ΔompA* mutant of *A. baumannii* in a transwell setting (6 hpi).

(f) LDH release assay of A549 cells infected with *A. baumannii* (wild-type ATCC 19606 or the *ΔompA* mutant) 24 hpi, with the addition of T5224 (100 μM). Cells were seeded in 3 different wells per group, Treatment and measurement were performed independently for each well. Experiments were repeated independently three times and similar results were obtained.

(g) GO analysis of the significantly differentially regulated proteins presented in Figure 3G. Enrichment P-value are derived using the hypergeometric test. To correct for multiple testing, False Discovery Rate (FDR) is calculated using the Benjamini-Hochberg method.

(a, b ,c ,f) Data are presented as mean values +/- SD, P-values by unpaired two-tailed t-test.

(d, e) The experiments were repeated three times with similar results obtained.

Source data are provided in the Source Data file.

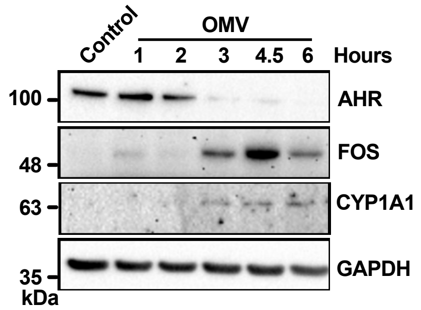

Supplementary Figure 4. Dynamics of FOS induction and AHR activation by OMVs, related to Figure 4

Time course experiment of A549 cells treated with *A. baumannii* OMVs (100 µg/mL). The experiments were repeated three times with similar results obtained. Source data are provided in the Source Data file.

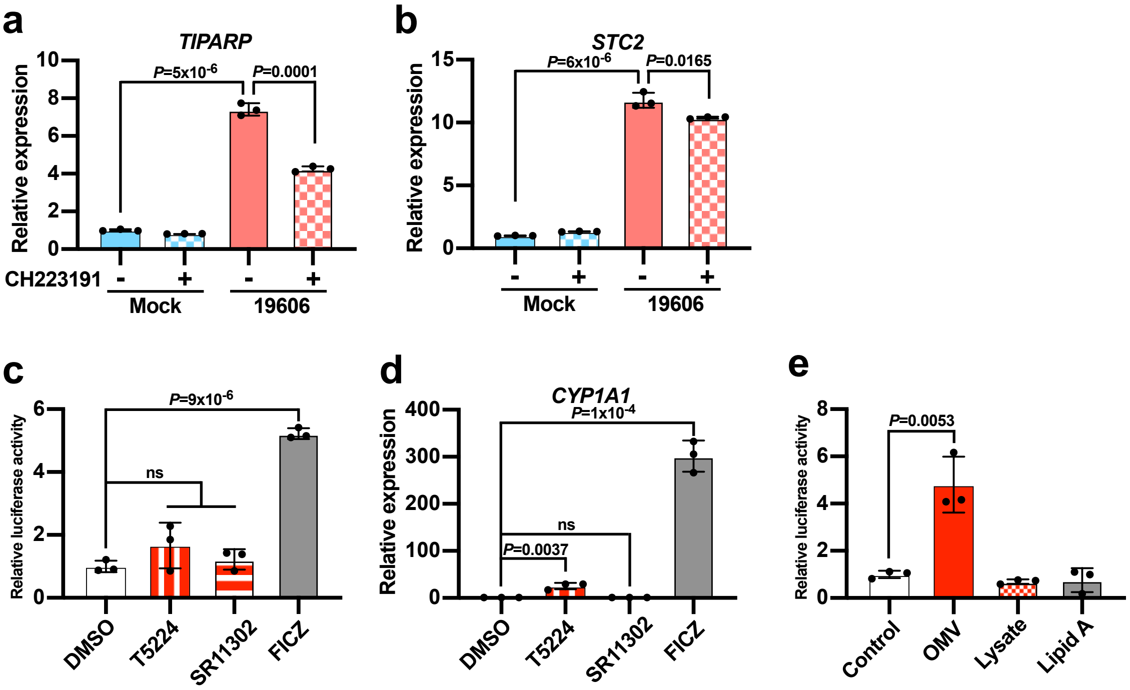

Supplementary Figure 5. *A. baumannii* infections induce expression of AHR target genes, related to Figure 5.

(a,b) Quantification of *TIPARP* and *STC2* transcript levels by qRT-PCR in A549 cells infected with *A. baumannii* ATCC 19606 (3 hpi). CH223191 (10 μM) was added to inhibit AHR. n = 3 independent experiments.

(c) AHR activity reporter (XRE-Luc) assay in A549 cells exposed to T5224 (100 μM), SR11302 (10 μM) or FICZ (1 μM).

(d) Quantification of *CYP1A1* transcipt levels by qRT-PCR in A549 cells treated with T5224 (100 μM), SR11302 (10 μM) or FICZ (1 μM). n = 3 independent experiments.

(e) AHR activity reporter (XRE-Luc) assay in A549 OMVs (20 μg/mL), ATCC 19606 lysate or purified lipid A for 3 hours.

(c, e) Cells were seeded in 3 different wells per group, Treatment and measurement were performed independently for each well. Experiments were repeated independently three times and similar results were obtained.

(a, b, c, d, e) Data are presented as mean values +/- SD, P-values by unpaired two-tailed t-test.

Source data are provided in the Source Data file.

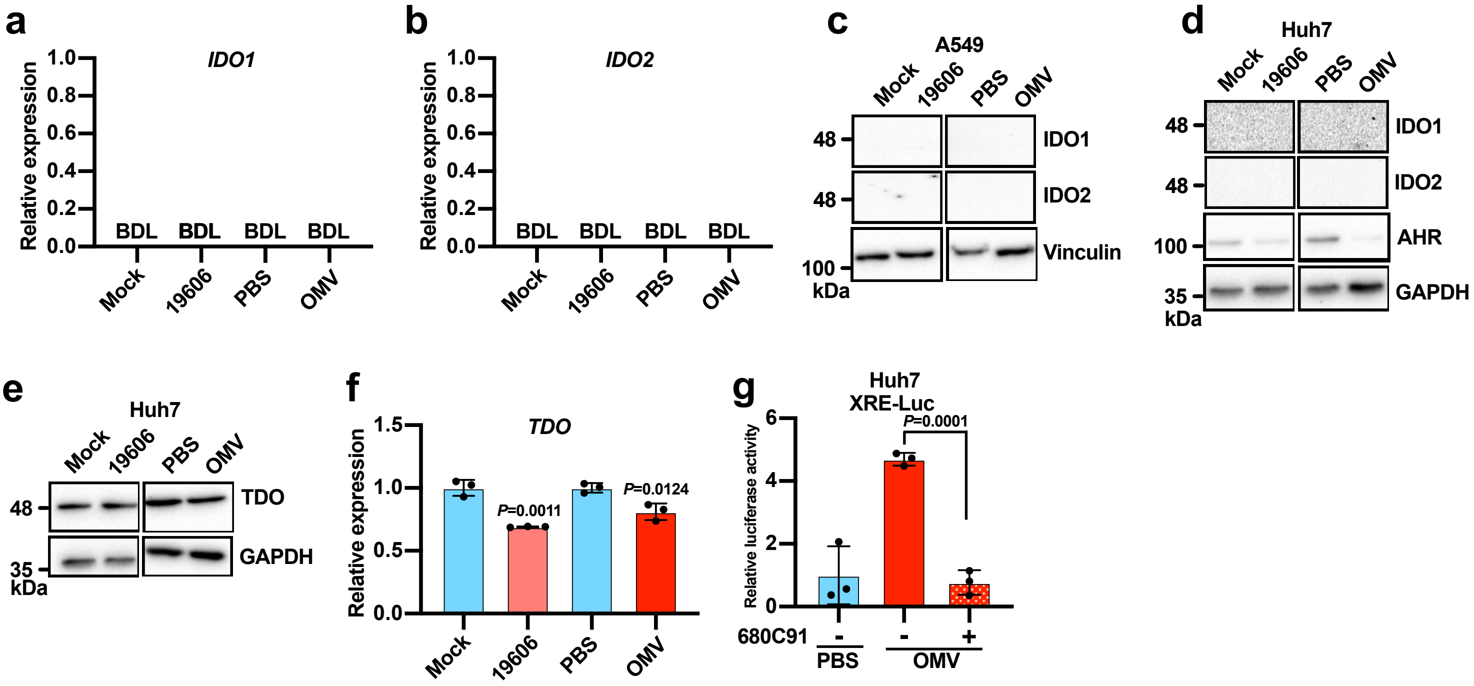

Supplementary Figure 6. TDO, but not IDOs, mediates the responses to OMVs, related to Figure 6.

(a,b,f) Quantification of *IDO1*, *IDO2* and *TDO* transcript levels by qRT-PCR in A549 cells infected with *A. baumannii* ATCC 19606 or exposed to its OMVs (100 µg/mL) for 3 h. n = 3 independent experiments.

(c,d,e) Western blot analysis of A549 cells (c) or Huh7 cells (d, e) after exposure to *A. baumannii* ATCC 19606 or its OMVs (100 µg/mL) for 3 h. The experiments were repeated three times with similar results obtained.

(g) AHR activity reporter (XRE-Luc) assay in Huh7 cells exposed to *A. baumannii* OMVs (20 µg/mL) and TDO inhibitor 680C91 (1 µM) for 3 h. Cells were seeded in 3 different wells per group, Treatment and measurement were performed independently to each well Experiments were repeated independently three times and similar results were obtained.

(a, b, f, g) Data are presented as mean values +/- SD, P-values by unpaired two-tailed t-test.

BDL: below detection limit. Source data are provided in the Source Data file.

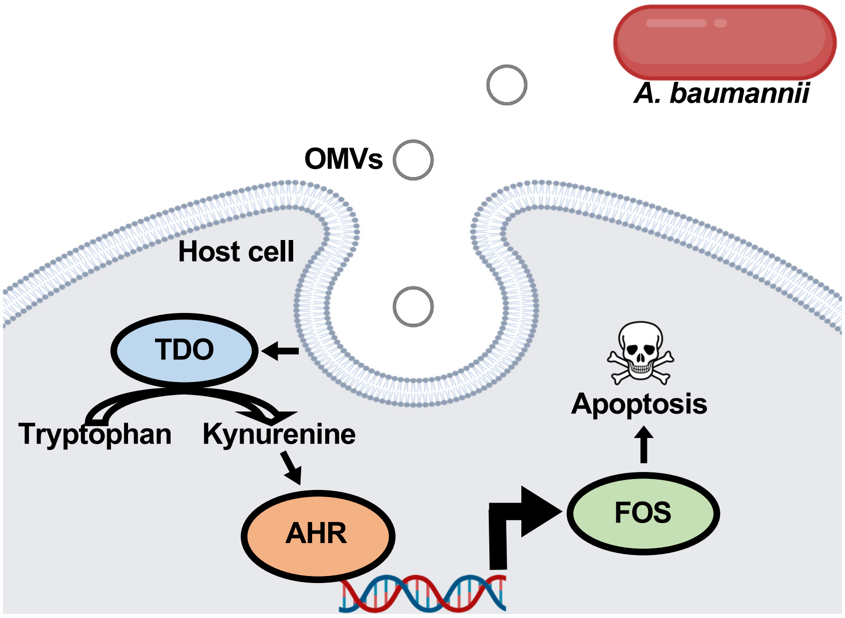

Supplementary Figure 7. Model of the roles of AHR and FOS in cytotoxicity induced by *A. baumannii* .

Created with BioRender.com released under a Creative Commons Attribution-NonCommercial-NoDerivs 4.0 International license (https://creativecommons.org/licenses/by-nc-nd/4.0/deed.en).
