# Supplementary material for: The aryl hydrocarbon receptor and FOS mediate cytotoxicity induced by Acinetobacter baumannii
Source: Nat Commun. 2024 Sep 11;15:7939. doi: 10.1038/s41467-024-52118-7 (PMC11390868; doi:10.1038/s41467-024-52118-7)
Supplement: Supplementary file 3 — Description of Additional Supplementary Files [file 41467_2024_52118_MOESM3_ESM.pdf]

Description of Additional Supplementary Files

Supplementary Data 1: Proteomic comparison of infected and mock-treated A549 cells. Related to figure 1. Only statistically significant results are shown. Mock=Mock-treated. WT=infected. Abundence and difference are presented in Log_2 scale.

Supplementary Data 2: Proteomic comparison of OMV and mock-treated A549 cells. Related to figure 3. Only statistically significant results are shown. PBS=Control (PBS-treated for 3 hours). 3h=OMV-treated for 3 hours. Abundence and difference are presented in Log_2 scale.
