# Supplementary material for: The aryl hydrocarbon receptor and FOS mediate cytotoxicity induced by Acinetobacter baumannii
Source: Nat Commun. 2024 Sep 11;15:7939. doi: 10.1038/s41467-024-52118-7 (PMC11390868; doi:10.1038/s41467-024-52118-7)
Supplement: Supplementary file 6 — Oligonucleotides [file 41467_2024_52118_MOESM6_ESM.pdf]

Oligonucleotides		
GAPDH qPCR primer F<br><br>AATCCCATCACCATCTTCCA	Sigma-Aldrich	
GAPDH qPCR primer R<br><br>TGGACTCCACGACGTACTCA	Sigma-Aldrich	
FOS qPCR primer F<br><br>GCCTCTCTTACTACCACTCACC	Sigma-Aldrich	
FOS qPCR primer R<br><br>AGATGGCAGTGACCGTGGGAAT	Sigma-Aldrich	
IL6 qPCR primer F<br><br>CAACCTGAACCTTCCAAAGATG	Sigma-Aldrich	
IL6 qPCR primer R<br><br>ACCTCAAACTCCAAAAGACCAG	Sigma-Aldrich	
IL8 qPCR primer F<br><br>TCCTGATTTCTGCAGCTCTG	Sigma-Aldrich	
IL8 qPCR primer R<br><br>GTCCACTCTCAATCACTCTCAG	Sigma-Aldrich	
IFNα qPCR primer F<br><br>TGGGCTGTGATCTGCCTCAAAC	Sigma-Aldrich	
IFNα qPCR primer R<br><br>CAGCCTTTTGGAACTGGTTGCC	Sigma-Aldrich	
TNFα qPCR primer F<br><br>CTCTTCTGCCTGCTGCACTTTG	Sigma-Aldrich	
TNFα qPCR primer R<br><br>ATGGGCTACAGGCTTGTCACTC	Sigma-Aldrich	
CCL2 qPCR primer F<br><br>AGAATCACCAGCAGCAAGTGTCC	Sigma-Aldrich	

CCL2 qPCR primer R<br><br>TCCTGAACCCACTTCTGCTTGG	Sigma-Aldrich	
CDK5 qPCR primer F<br><br>CCACAACATCCCTGGTGAACGT	Sigma-Aldrich	
CDK5 qPCR primer R<br><br>CCTCTTCTGCTGAGATACGCTG	Sigma-Aldrich	
BIRC3 qPCR primer F<br><br>GCTTTTGCTGTGATGGTGGACTC	Sigma-Aldrich	
BIRC3 qPCR primer R<br><br>CTTGACGGATGAACTCCTGTCC	Sigma-Aldrich	
BCL3 qPCR primer F<br><br>GAACACCGAGTGCCAAGAAACC	Sigma-Aldrich	
BCL3 qPCR primer R<br><br>GCTAAGGCTGTTGTTTTCCACGG	Sigma-Aldrich	
BNIP3 qPCR primer F<br><br>TCAGCATGAGGAACACGAGCGT	Sigma-Aldrich	
BNIP3 qPCR primer R<br><br>GAGGTTGTCAGACGCCTTCCAA	Sigma-Aldrich	
BIM qPCR primer F<br><br>CAAGAGTTGCGGCGTATTGGAG	Sigma-Aldrich	
BIM qPCR primer R<br><br>ACACCAGGCGGACAATGTAACG	Sigma-Aldrich	
FOS promoter set 1 F<br><br>CCGTGACGTTTACACTCATTC	Sigma-Aldrich	
FOS promoter set 1 R<br><br>CTTGGCTTCTCAGATGCTC	Sigma-Aldrich	
FOS promoter set 2 F<br><br>GCATCTGAGAAGCCAAGAC	Sigma-Aldrich	

FOS promoter set 2 R<br><br>GAAGCCCGAGAACATCATC	Sigma-Aldrich	
FOS promoter set 3 F<br><br>TGAGAAGCCAAGACTGAGCC	Sigma-Aldrich	
FOS promoter set 3 R<br><br>CGTTGAAGCCCGAGAACATC	Sigma-Aldrich	
CYP1A1 qPCR primer F<br><br>GATTGAGCACTGTCAGGAGAAGC	Sigma-Aldrich	
CYP1A1 qPCR primer R<br><br>ATGAGGCTCCAGGAGATAGCAG	Sigma-Aldrich	
CYP1B1 qPCR primer F<br><br>GCCACTATCACTGACATCTTCGG	Sigma-Aldrich	
CYP1B1 qPCR primer R<br><br>CACGACCTGATCCAATTCTGCC	Sigma-Aldrich	
TIPARP qPCR primer F<br><br>GATTCTCAGGAGCACTTGGAAAG	Sigma-Aldrich	
TIPARP qPCR primer R<br><br>TGGTGTGGACAGCCTTCGTAGT	Sigma-Aldrich	
AHRR qPCR primer F<br><br>CACCAGTCTGTGCGAATCGGAA	Sigma-Aldrich	
AHRR qPCR primer R<br><br>CAGTCTGTTCCCTGAGCACCAA	Sigma-Aldrich	
STC2 qPCR primer F<br><br>GCATGACTTTTCTGCACAACGCT	Sigma-Aldrich	
STC2 qPCR primer R<br><br>GGCTTATGCAGCCGAACCTGTG	Sigma-Aldrich	
IDO1 qPCR primer F<br><br>GCCTGATCTCATAGAGTCTGGC	Sigma-Aldrich	

IDO1 qPCR primer R<br><br>TGCATCCCAGAACTAGACGTGC	Sigma-Aldrich	
IDO2 qPCR primer F<br><br>GTTATGTCTGGCAGGAAGGAGAG	Sigma-Aldrich	
IDO2 qPCR primer R<br><br>GTCCAGTTCGTCAGCACCAAGT	Sigma-Aldrich	
TDO qPCR primer F<br><br>CAGGTGCCTTTTCAGTTGCTGAC	Sigma-Aldrich	
TDO qPCR primer R<br><br>GTAGTGATAGCCTGAGGAACCAC	Sigma-Aldrich	
ON-TARGETplus Human AHR siRNA SMARTPool	Dharmacon™	Catalog ID:L-004990-00-0005
ON-TARGETplus Non-targeting Control Pool	Dharmacon™	Catalog ID:D-001810-10-50
