# Supplementary material for: The aryl hydrocarbon receptor and FOS mediate cytotoxicity induced by Acinetobacter baumannii
Source: Nat Commun. 2024 Sep 11;15:7939. doi: 10.1038/s41467-024-52118-7 (PMC11390868; doi:10.1038/s41467-024-52118-7)
Supplement: Supplementary file 7 — Reporting summary [file 41467_2024_52118_MOESM7_ESM.pdf]

nature portfolio

Corresponding author(s): Ivan Dikic

Last updated by author(s): 2024.07.18

Reporting Summary

Nature Portfolio wishes to improve the reproducibility of the work that we publish. This form provides structure for consistency and transparency in reporting. For further information on Nature Portfolio policies, see our Editorial Policies and the Editorial Policy Checklist.

Statistics

For all statistical analyses, confirm that the following items are present in the figure legend, table legend, main text, or Methods section.

n/a	Confirmed	
☐	☒	The exact sample size (*n*) for each experimental group/condition, given as a discrete number and unit of measurement
☐	☒	A statement on whether measurements were taken from distinct samples or whether the same sample was measured repeatedly
☐	☒	The statistical test(s) used AND whether they are one- or two-sided<br>*Only common tests should be described solely by name; describe more complex techniques in the Methods section.*
☐	☒	A description of all covariates tested
☐	☒	A description of any assumptions or corrections, such as tests of normality and adjustment for multiple comparisons
☐	☒	A full description of the statistical parameters including central tendency (e.g. means) or other basic estimates (e.g. regression coefficient) AND variation (e.g. standard deviation) or associated estimates of uncertainty (e.g. confidence intervals)
☐	☒	For null hypothesis testing, the test statistic (e.g. *F*, *t*, *r*) with confidence intervals, effect sizes, degrees of freedom and *P* value noted<br>*Give P values as exact values whenever suitable.*
☒	☐	For Bayesian analysis, information on the choice of priors and Markov chain Monte Carlo settings
☒	☐	For hierarchical and complex designs, identification of the appropriate level for tests and full reporting of outcomes
☐	☒	Estimates of effect sizes (e.g. Cohen's *d*, Pearson's *r*), indicating how they were calculated

Our web collection on statistics for biologists contains articles on many of the points above.

Software and code

Policy information about availability of computer code

Data collection	Biorad ImageLab 6.1<br>Thermo Scientific Xcalibur 4.4<br>Tecan i-Control 1.9<br>Bio-Rad CFX Maestro 2.0
Data analysis	GraphPad Prism v8<br>Proteome Discoverer v2.4<br>Perseus

For manuscripts utilizing custom algorithms or software that are central to the research but not yet described in published literature, software must be made available to editors and reviewers. We strongly encourage code deposition in a community repository (e.g. GitHub). See the Nature Portfolio guidelines for submitting code & software for further information.

Data

Policy information about availability of data

All manuscripts must include a data availability statement. This statement should provide the following information, where applicable:

- Accession codes, unique identifiers, or web links for publicly available datasets
- A description of any restrictions on data availability
- For clinical datasets or third party data, please ensure that the statement adheres to our policy

Uncropped blots and numerical values are provided as source files. The mass spectrometry proteomics data have been deposited to the ProteomeXchange Consortium via the PRIDE partner repository with the dataset identifier PXD053768.

Research involving human participants, their data, or biological material

Policy information about studies with human participants or human data. See also policy information about sex, gender (identity/presentation), and sexual orientation and race, ethnicity and racism.

Reporting on sex and gender	Not applicable
Reporting on race, ethnicity, or other socially relevant groupings	Not applicable
Population characteristics	Not applicable
Recruitment	Not applicable
Ethics oversight	Not applicable

Note that full information on the approval of the study protocol must also be provided in the manuscript.

Field-specific reporting

Please select the one below that is the best fit for your research. If you are not sure, read the appropriate sections before making your selection.

☒ Life sciences ☐ Behavioural & social sciences ☐ Ecological, evolutionary & environmental sciences

For a reference copy of the document with all sections, see nature.com/documents/nr-reporting-summary-flat.pdf

Life sciences study design

All studies must disclose on these points even when the disclosure is negative.

Sample size	10 mice per group were used for the survival assay.<br>3 to 5 replicates were used for mass spectrometry, qPCR and luciferase reporter assays.<br>Sample sizes were chosen based on common experimental practice to facilitate downstream statistical analysis.
Data exclusions	None.
Replication	At least 3 independent biological replicates were performed for each experiment.
Randomization	No randomization was required for this study.
Blinding	No blinding was required for this study.

Reporting for specific materials, systems and methods

We require information from authors about some types of materials, experimental systems and methods used in many studies. Here, indicate whether each material, system or method listed is relevant to your study. If you are not sure if a list item applies to your research, read the appropriate section before selecting a response.

Materials & experimental systems

n/a	Involved in the study	
☐	☒	Antibodies
☐	☒	Eukaryotic cell lines
☒	☐	Palaeontology and archaeology
☐	☒	Animals and other organisms
☒	☐	Clinical data
☒	☐	Dual use research of concern
☒	☐	Plants

Methods

n/a	Involved in the study	
☒	☐	ChIP-seq
☒	☐	Flow cytometry
☒	☐	MRI-based neuroimaging

Antibodies

Antibodies used

Cell signaling:
c-Fos (9F6) Rabbit mAb #2250 1:2000
GAPDH (14C10) Rabbit mAb #2118 1:5000
IκBα (44D4) Rabbit mAb #4812 1:2000
PARP Antibody #9542 1:2000
Caspase-9 Antibody (Human Specific) #9502 1:2000
β-Tubulin (9F3) Rabbit mAb #2128 1:5000

Sigma-Aldrich:
Monoclonal Anti-Vinculin antibody produced in mouse clone VIN-11-5 1:5000

Santa Cruz:
Anti-Ah Receptor Antibody (A-3): sc-133088 1:100
mouse IgG kappa binding protein (m-IgGκ BP) conjugated to Horseradish Peroxidase (HRP) sc-516102 1:5000

abcam:
Recombinant Anti-TDO2/TDO antibody (ab281571) 1:2000
Recombinant Anti-Indoleamine 2, 3-dioxygenase antibody (ab211017) 1:2000

ThermoFisher Scientific:
IDO2 Monoclonal Antibody (OTI2B9) 1:2000
CYP1A1 Polyclonal Antibody (# PA5-15213) 1:2000

Agilent:
Goat Anti-Rabbit Immunoglobulins/HRP P0448 1:5000

Validation

All antibodies are commercially available and validated by the manufacturers.

Eukaryotic cell lines

Policy information about cell lines and Sex and Gender in Research

Cell line source(s)

A549, HEK293T and RAW264.7 were obtained from ATCC.
Human monocyte derived macrophages (MDM) were differentiated at our labs. Blood was obtained from male donors at a local blood donation center.
Huh-7 cells were a gift from Sandra Ciesek lab (Goethe University Frankfurt).

Authentication

Not required.

Mycoplasma contamination

Cells were negative for mycoplasma contamination.

Commonly misidentified lines
(See ICLAC register)

None.

Animals and other research organisms

Policy information about studies involving animals; ARRIVE guidelines recommended for reporting animal research, and Sex and Gender in Research

Laboratory animals

Mus musculus BALB/c female. 5-6 weeks of age.

Wild animals

none.

Reporting on sex	Only female animals were used for experimental consistency.
Field-collected samples	none.
Ethics oversight	The animal studies were reviewed and approved by the ethic committee PDST IACUC (Pharmacology Discovery Services Taiwan Institutional Animal Care and Use Committee).

Note that full information on the approval of the study protocol must also be provided in the manuscript.

Plants

Seed stocks	*Report on the source of all seed stocks or other plant material used. If applicable, state the seed stock centre and catalogue number. If plant specimens were collected from the field, describe the collection location, date and sampling procedures.*
Novel plant genotypes	*Describe the methods by which all novel plant genotypes were produced. This includes those generated by transgenic approaches, gene editing, chemical/radiation-based mutagenesis and hybridization. For transgenic lines, describe the transformation method, the number of independent lines analyzed and the generation upon which experiments were performed. For gene-edited lines, describe the editor used, the endogenous sequence targeted for editing, the targeting guide RNA sequence (if applicable) and how the editor was applied.*
Authentication	*Describe any authentication procedures for each seed stock used or novel genotype generated. Describe any experiments used to assess the effect of a mutation and, where applicable, how potential secondary effects (e.g. second site T-DNA insertions, mosiacism, off-target gene editing) were examined.*
